# Laser interstitial thermal therapy (LITT) for pediatric patients affected by intracranial tumors

**DOI:** 10.3389/fneur.2023.1120286

**Published:** 2023-04-20

**Authors:** Barbara Spacca, Marco Di Maurizio, Manuela Grandoni, Sara Tempesti, Lorenzo Genitori

**Affiliations:** ^1^Neurosurgery Unit, Meyer Children’s Hospital IRCCS, Florence, Italy; ^2^Radiology Unit, Meyer Children’s Hospital IRCCS, Florence, Italy

**Keywords:** LITT, laser, laser interstitial thermal therapy, brain tumor, pediatric neuro-oncology

## Abstract

**Introduction:**

The surgical treatment of brain tumors has evolved over time, offering different strategies tailored to patients and their specific lesions. Among these strategies, Laser Interstitial Thermal Therapy (LITT) is one of the most recent advances in pediatric neurooncological surgery, and its results and evolution are still under assessment.

**Methods:**

We retrospectively analyzed data from six pediatric patients with deep-seated brain tumors treated with LITT at a single center between November 2019 and June 2022. A total of four patients underwent a stereotaxic biopsy during the same operating session. The indications and preparation for LITT, technical issues, clinical and radiological follow-up, impact on quality of life, and oncological treatment are discussed.

**Results:**

The mean patient age eight years (ranging from 2 to 11 years). The lesion was thalamic in four patients, thalamo-peduncular in one, and occipital posterior periventricular in one. In total, two patients had been previously diagnosed with low-grade glioma (LGG). Biopsies revealed LGG in two patients, ganglioglioma grade I in one, and diffuse high-grade glioma (HGG) in one. Postoperatively, two patients presented with transient motor deficits. The mean follow-up period was 17 months (ranging from 5 to 32 months). Radiological follow-up showed a progressive reduction of the tumor in patients with LGG.

**Conclusion:**

Laser interstitial thermal therapy is a promising, minimally invasive treatment for deep-seated tumors in children. The results of lesion reduction appear to be relevant in LGGs and continue over time. It can be used as an alternative treatment for tumors located at sites that are difficult to access surgically or where other standard treatment options have failed.

## 1. Introduction

Brain tumors, either deep-sited or close to eloquent areas, continue to be challenging to treat and can be considered either not amenable to surgical treatment or treatable with a very high risk of post-surgical neurological complications. Laser interstitial thermal therapy (LITT) is one of the most recent surgical techniques introduced for the neurosurgery of brain tumors. Its advantages are mainly related to its minimal invasiveness and the possibility of applying it to a range of neurosurgical pathologies, even in areas that are difficult to reach with microsurgery. Its use in pediatric neurosurgery has developed over the past decade, with the first case of LITT being used in the treatment of a brain tumor in a child reported in 2011 (1). Starting from this report, the use of LITT in children with brain tumors has grown, but it is still limited; available publications are relatively scarce, and series are limited. Moreover, the use of the stereotactic technique in children is limited in very young people by the thickness of the skull. Tumor treatment in children can be contentious in terms of the risk of late brain damage caused by chemotherapy and radiotherapy, especially in very young children and children with deep-seated non-resectable lesions and aggressive behavior. We reviewed our initial experience with LITT in children for the treatment of brain tumors since 2019 and analyzed the results according to pertinent literature and a multistep strategy of treatment.

## 2. Materials and methods

**Figure 1:**
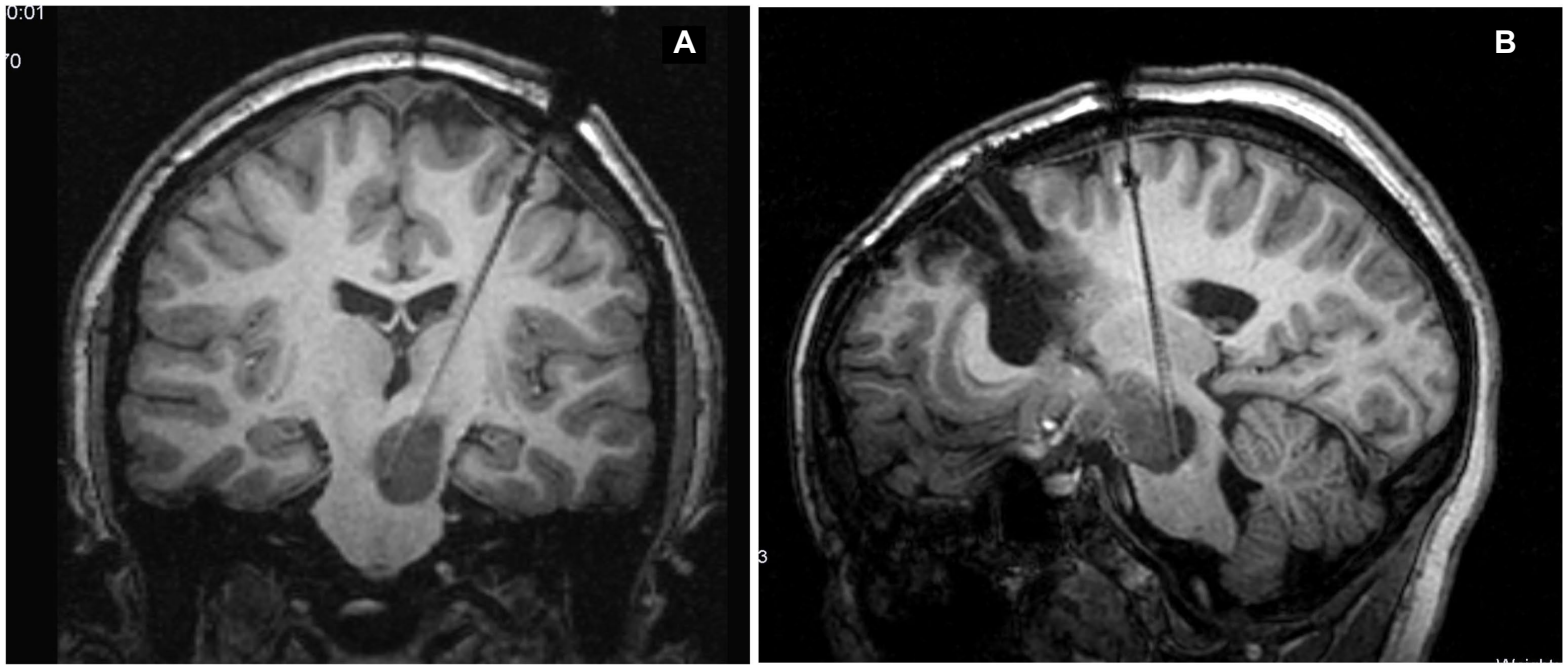
Slices chosen to be transferred to the Visualase system that are along the catheter trajectory **(A)** and orthogonal in position **(B)**.

All patients underwent surgery under general anesthesia and orotracheal intubation. As detailed later, three different stereotactic techniques were used for laser fiber implant and biopsy in four patients. After the first case performed with the Vertek® Medtronic System, a robotic technique was chosen to improve trajectory accuracy. In the last case, a pinless neuronavigated technique was chosen due to the thinnes of the skull. In all cases, the accuracy of the target was adequate for LITT treatment and to obtain a diagnostic biopsy when required. A 10 mm laser diffusing fiber (LDF) was used in all cases. The surgical plan was decided with the StealthStation S8® Medtronic Navigation System with magnetic resonance imaging (MRI) obtained the day before surgery in all but case 6, where MRI was obtained before 6 days. A minimal hair shave was performed at the entry site to allow the skin incision required according to the chosen stereotactic technique, with a minimum of 1 cm and a maximum of 2.5 cm. The burr-hole was obtained with a 3.2 twist drill in all cases. The LDF was then inserted into the target according to the preplanned trajectory and fixed to the skull with the specific titanium bolt provided. Patients were then transferred to the MRI suite (3 T, Philips Smart Path Stream), and the initial sequences aimed to confirm the trajectory, and the target were as planned. Then, the laser applicator was connected to the Visualase® Medtronic System, and images were angled to incorporate the whole catheter along its trajectory in a slice, while the second image transferred to the Visualase® machine was orthogonal to the trajectory and centered on the target (Figure 1). Target thermal protection points were then set to help preserve the thermal damage to the surrounding structures (2). The neurosurgeon and radiologist worked as a team following thermographic acquisition during the on and off time of the laser. After any session and before starting a new one, there is an interval to reset the temperature to physiological values on the thermal reference points and allow an estimation of the damage obtained. The tip of the LDF is retracted when needed according to the volume of the lesions and the extension of the thermal damage. The correct position was then rechecked using an anatomical MRI. At the end of the procedure, once the maximum extent of damage was determined, a final standard MRI acquisition was obtained with diffusion and post-contrast enhancement to evaluate the early effects and possible complications (3). The patients were then transferred to the operating room for LDF, bolt removal, and awakening. None of the patients required intensive care after the procedure, and all were transferred to the neurosurgical ward.

## 3. Case series

### 3.1. Case 1

**Figure 2:**
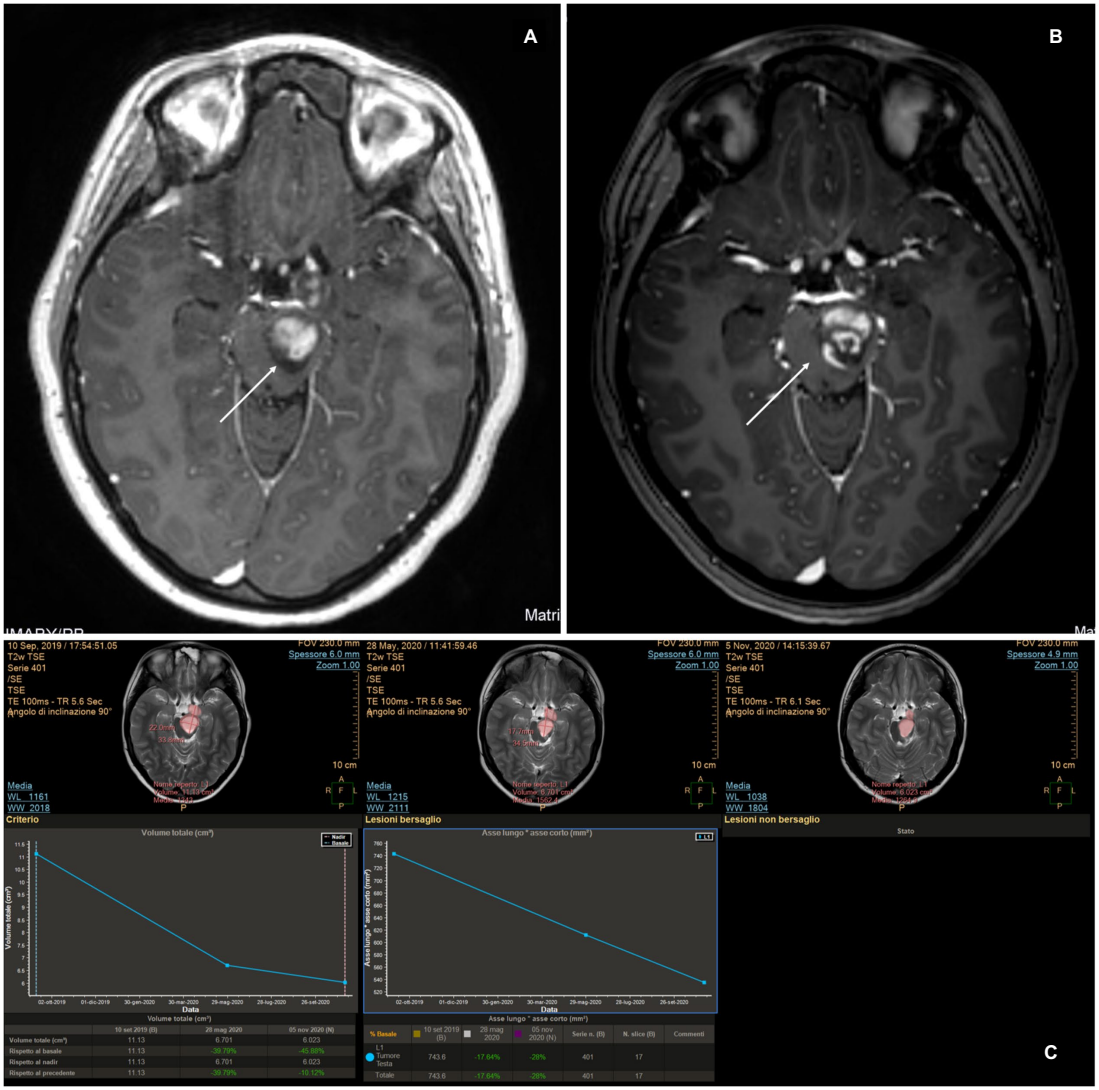
**(A)** Pre-operative magnetic resonance imaging (MRI). **(B)** MRI at 48 h after magnetic resonance-guided laser interstitial thermal therapy. A change in the post-contrast enhancement is made evident, compared to the pre-operative scan in panel **(A)**. A lack of enhancement in the central portion of the tumor where the thermal effect induced coagulation and necrosis can be observed. **(C)** MRI examined with the MITT Intellispace Portal. At 12 months after surgery, the tumor was found to have reduced by 46% in volume compared to pre-operative imaging and by 11% compared to the scan 6 months after surgery.

**Figure 3:**
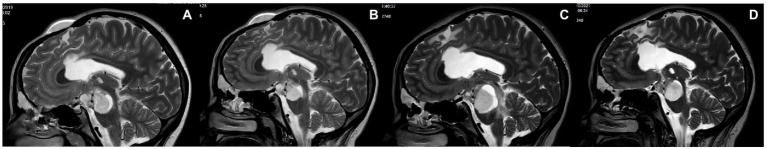
**(A)** Pre-operative MRI. **(B)** Follow-up MRI 6 months after LITT. Both the treated ponto-mesencephalic portion and the anterior nodular portion of the tumor are reduced. **(C)** Follow-up MRI 18 months after LITT. The tumor regrew starting from the treated site and presented a relevant perilesional edema. **(D)** Follow-up MRI 23 months after LITT during chemotherapy treatment. Both the cystic and the solid portions of the tumor appear reduced in size.

An 11-year-old girl was diagnosed at 5 years of age with a left-sided thalamo-peduncular tumor after the onset of right hemiparesis. Surgery had been performed in two different centers with an overall partial resection. One surgery was complicated with hydrocephalus. The histology of the patient was pilocytic astrocytoma. Hemiparesis was partially improved. After 5 years, she presented with pain, and MRI showed disease progression. She was started on chemotherapy (carboplatin and etoposide), but after 6 months, the tumor size increased, and LITT was offered. Previous surgeries were impone to plan the fiber trajectory for LITT according to the previous craniotomy/craniectomy location, possible bone defects, and residual gliotic and/or cystic areas from the previous surgical accesses. Surgery was performed using the Vertek® Medtronic System. No biopsy was attempted at that time, and the target was set in the middle of the residual tumor. The laser was switched on for a total of 14 min, with a mean dose of 4.54 W (min 2.25, max 8.55). Previous resections did not affect the efficacy of LITT. The patient experienced no complications (Figure 2). The tumor showed a progressive reduction in volume over the first 6 months postoperatively but started to increase in size again at the 1-year follow-up. A further increase in size was evident at 18 months follow-up (Figure 3), and new treatment was initiated (bevacizumab and irinotecan). No clinical deterioration was noted, and at the last follow-up, 32 months after LITT, the lesion was stabilized with chemotherapy.

### 3.2. Case 2

An 8-year-old boy had been diagnosed 8 months earlier with a left thalamic tumor extending to the mesencephalic and pontine regions. He had been operated on elsewhere through a subtemporal approach and partial resection, which was complicated by right-sided hemiparesis. After surgery, the patient developed hydrocephalus and was treated with a ventriculoperitoneal (VP) shunt. This was further complicated by an extradural hematoma related to excessive drainage that required surgical evacuation. The histology of the patient showed pilocytic astrocytoma. MRI taken 6 months after surgery revealed tumor progression, and the family sought a second opinion. On presentation, he was wheelchair-bound with severe right-sided facio-brachio-crural hemiparesis. Different treatment options were discussed with the family. Finally, LITT was agreed upon because the parents were scared to undergo further conventional surgery. The laser was switched on for 14 min, with a mean dose of 8.7 W (minimum 3 and maximum 12.9 W). Because of the dimension of the tumor, the safest trajectory was chosen toward the middle of the tumor with Autoguide Medtronic®. After surgery, the patient presented with clinical deterioration, a headache, and worsened hemiparesis that appeared to be related to a change in the hydrocephalus. The patient was then treated with an endoscopic procedure consisting of the marsupialization of the cystic tumor and a third ventriculostomy plus removal of the VP shunt. From the first postoperative day after the endoscopy, the headache got resolved, and with intensive rehabilitation, the right hemiparesis got improved. At the last follow-up, the patient could walk with a right foot brace without the need for help. His right arm and leg were 4/5, he had residual VII cranial nerve palsy that was grade II on the House Brackman scale, and he attended school regularly with no learning difficulties. Follow-up MRI revealed a surprisingly progressive reduction in the mass, which continued after 21 months. The volume reduction between the pre-operative and last follow-up MRI scans was evaluated using MITT Intellispace Portal imaging and resulted in a 35.2% reduction of the solid portion of the tumor. The patient did not receive any adjuvant therapy.

### 3.3. Case 3

**Figure 4:**
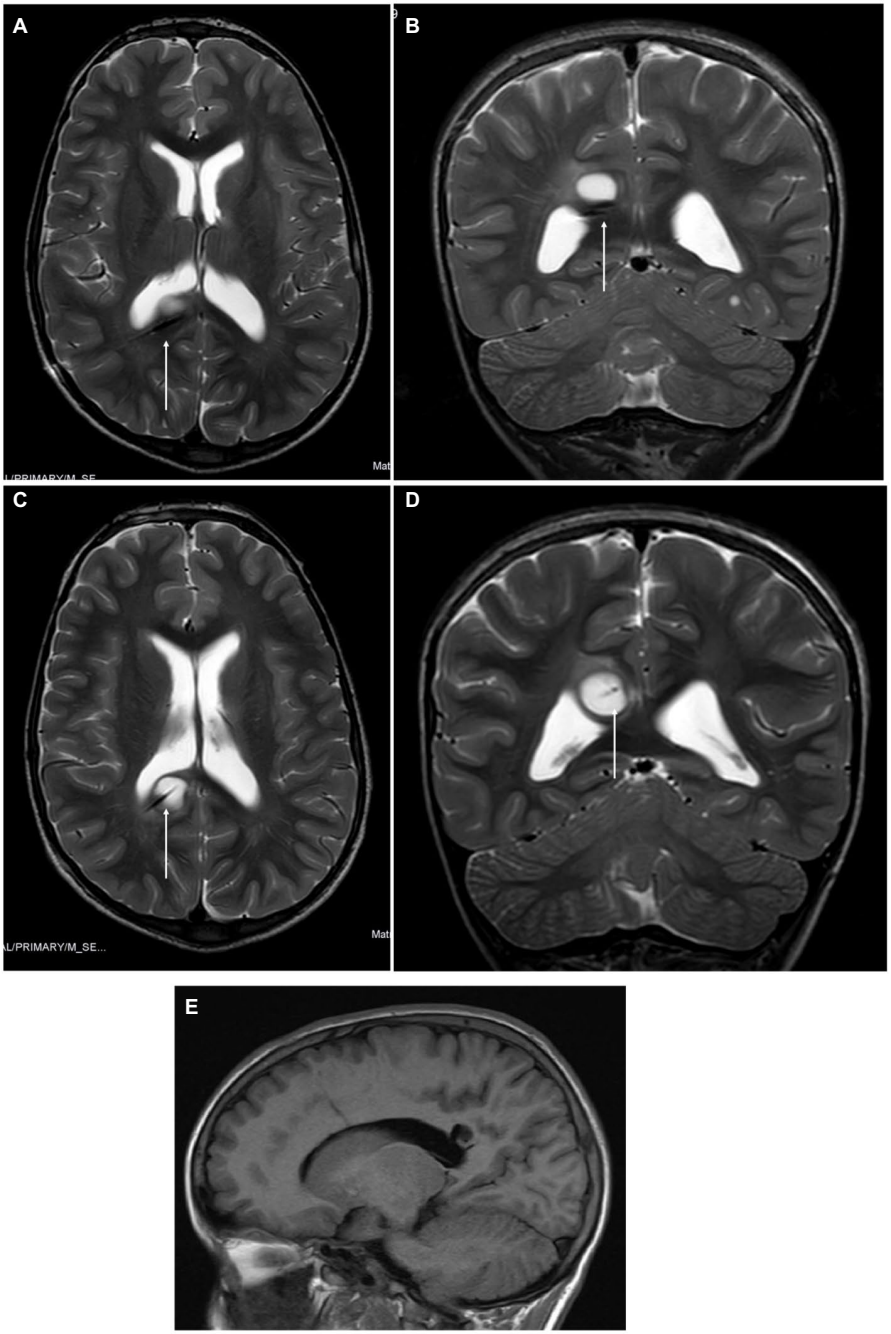
**(A,B)** Magnetic resonance imaging (MRI) revealed a suboptimal position of the tip of the laser diffusing fiber (LDF) on axial and coronal slices T2 sequences. No bleeding was detected. **(C,D)** MRI after repositioning the LDF before starting the procedure, with the tip on the middle of the right occipito-parietal tumor. **(E)** At the 21-month follow-up, the MRI scan revealed that the tumor had a cystic appearance and was filled with CSF. There was no evidence of contrast enhancement.

A 9-year-old boy with NF1 genetically confirmed was previously operated on for hydrocephalus with endoscopic third ventriculostomy. During routine follow-up, the patient developed a right parieto-occipital mass of increasing size. A biopsy was required for oncological purposes, and LITT treatment at the same time was discussed with his family. The procedure was carried out with the help of Autoguide® Medtronic. The histology of the patient showed low-grade glioma (LGG). Initially, the positioning of the laser fiber was suboptimal (Figures 4A–D), and the procedure needed to be reperformed. The procedure was then carried out uneventfully. The laser was switched on for 7 min with a mean dose of 4.2 W (minimum 3 and maximum 5.7 W). The patient was neurologically intact at the 21-month follow-up with no adjuvant therapy. The MRI of the tumor revealed complete cystic evolution and appeared as a cyst filled with cerebrospinal fluid (Figure 4E).

### 3.4. Case 4

**Figure 5:**
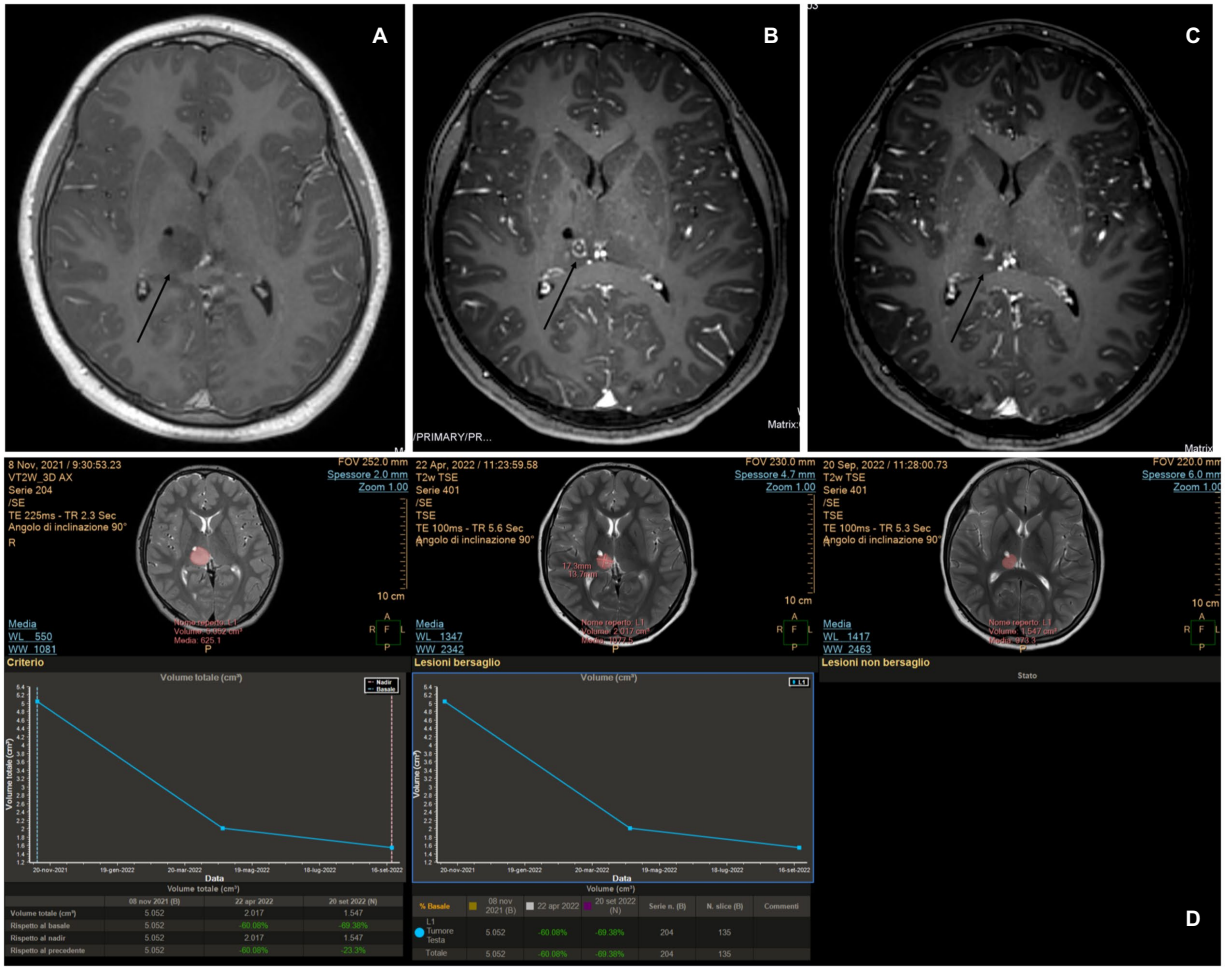
**(A)** Pre-operative magnetic resonance imaging (MRI) showing a right thalamic tumor with poor enhancement. **(B)** MRI obtained at 5 months revealed persistent blood–brain barrier damage, as suggested by the enhancement at the site where the tip of the laser diffusing fiber was located, and a surrounding ring enhancement on the border of the cone produced by the ablation thermal power exerted during the procedure. **(C)** MRI taken 10 months after the procedure still showed evidence of central enhancement. **(D)** MITT Intellispace Portal imaging revealed a volume reduction of 60% at 5 months and an overall reduction of 69% at 10 months.

An 11-year-old boy was diagnosed at the age of 2 years with an autism spectrum disorder. At that time, the MRI findings were unremarkable. At the age of 10 years, because of worsening behavior, a new head MRI was performed that revealed a right thalamic tumor. The patient underwent a stereotactic biopsy, which revealed LGG. At the 1-year follow-up, the tumor size had increased. Different treatment options were discussed with the patient’s family, and LITT treatment was agreed upon. Autoguide® Medtronic was used to place the fiber. The procedure was uneventful. The laser was switched on for a total of 9 min, and the mean power used was 6.7 W (minimum 4.2 and maximum 9.7 W). Postoperatively, the patient presented with left-hand weakness on day 1 and was started on steroids. He recovered completely after 2 months. Adjuvant therapy was not administered. At the 1-year follow-up, the tumor was seen to have shrunk (Figure 5).

### 3.5. Case 5

**Figure 6:**
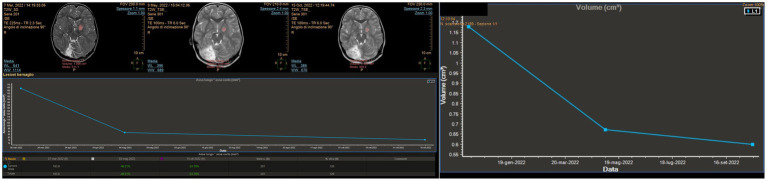
MITT Intellispace Portal imaging elaboration of magnetic resonance imaging shows a volumetric reduction of the known left thalamic tumor by more than 50% 7 months after surgery. The reduction of the proportion between the long and short axis is 54%.

An 8-year-old girl was previously diagnosed at the age of 2 years with a posterior fossa tumor and hydrocephalus. At that time, the patient underwent a third ventriculostomy and tumor resection. The histology of the patient showed medulloblastoma with dissemination to the cerebral spinal fluid. Treatment with chemotherapy and radiotherapy (AIEOP SNC infant high risk for metastatic disease protocol) and autologous bone marrow transplant was completed in 2016. At the end of 2020, a new lesion was noted on a follow-up MRI of the left thalamus. The lesion was stable but started to grow 1 year later. Positron emission tomography revealed an active metabolism. The patient remained neurologically intact. LITT and biopsy were performed in March 2022. The surgery was uneventful, and the patient was discharged on postoperative day 2. Autoguide® Medtronic was used for both the biopsy and fiber insertion. The histology of the patient showed gangliocytoma. The mean laser dose was 4.9 W (minimum 3.15 W and maximum 8.10 W), and the laser was switched on for 9 min. Further therapy was considered to target the molecular results but has not yet been administered. Follow-ups at 2 and 6 months showed a progressive reduction of the tumor (Figure 6).

### 3.6. Case 6

**Figure 7:**
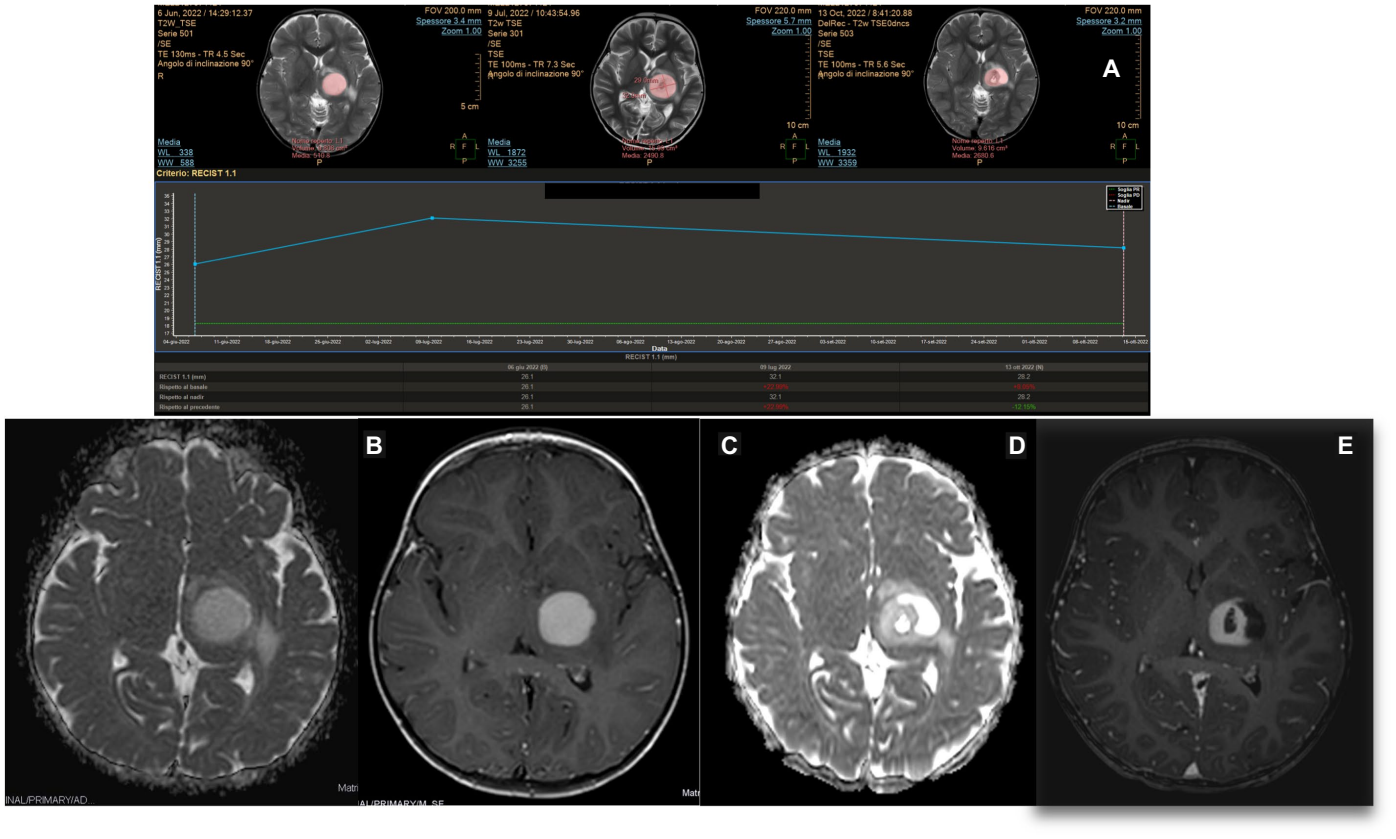
**(A)** MITT Intellispace Portal imaging examination. After 1 month of laser interstitial thermal therapy, when chemotherapy had not yet started, the tumor on diffusion (d) was 22% bigger because of intralesional edema. At 4 months, it was reduced by an overall 22%. **(B,C)** Pre-operative magnetic resonance imaging (MRI) on ADC and T1 TFE 3D post-enhancement imaging. **(D,E)** ADC and post-contrast-enhancement MRI obtained 5 months after surgery and after four cycles of chemotherapy demonstrated a cystic evolution of the tumor, with a clear reduction of the contrast enhancement and the involution of the central necrotic part.

A 2-year-old girl developed left hemiparesis and was diagnosed with a right thalamic space-occupying lesion. Biopsy and LITT were performed using the same procedures. Because the skull thickness was estimated at 4 mm, pins were not considered safe, and the biopsy and laser insertion were performed with Navigus® Medtronic. The level of accuracy was satisfactory in both (4, 5). The biopsy revealed HGG. The laser was switched on for 19 min, and the mean laser dose was 4.7 W (minimum 3.10 W and maximum 7 W). The patient had transient worsening of hemiparesis, but at 1 month, she had improved even compared to the pre-operative status. The patient was started on chemotherapy (AIEOP SNC infant). A 1-month scan revealed a swollen lesion; however, the treatment had not yet started. MRI after the fourth cycle of therapy at a 4-month follow-up showed a significant reduction in the tumor size (Figure 7). Because of her age, she cannot receive radiotherapy, and she will be scheduled for further surgery after the next MRI if the tumor is still evident and before the completion of therapy.

## 4. Results

Results are summarized in Table 1. A total of six patients, three boys and three girls, were treated for deep-seated tumors with LITT. LITT was the first surgery in four patients, while two patients had already undergone previous surgery.

**Table 1:** The table summarizes the results.

Case	Age, sex	Location	Histology	Previous surgery	Biopsy	Complications	Chemotherapy	Follow up (months)	Total volume percentage decrease compared to baseline
1	11, F	Thalamo-peduncular	Pilocytic astrocytoma	Open craniotomy	No		Yes – 1 year later	32	45,88%^1^
2	8, M	Thalamo-mesencephalic	Pilocytic astrocytoma	Open craniotomy	No		No	21	35.23%^2^
3	9, M	periventricular parieto-occipital	LGG	No	Yes		No	21	CSF filled cystic evolution
4	11, M	Thalamus	LGG	Robotic biopsy	No	Transient weakness	No	12	69,38%
5	8, F	Thalamus	Gangliocytoma	No	Yes		No	6	53,79%
6	2, F	Thalamus	HGG	No	Yes	Transient weakness	yes	4	12,15%

Two patients were started on chemotherapy. ^1^The overall reduction is referred to the volume at 32 months after LITT and at 14 months after the regrowth and start of chemotherapy. ^2^The volume reduction is referred to the solid portion of the tumor. No permanent complications were recorded.

Mortality was *nihil*, and morbidity consisted of two cases of transient weakness in the thalamic lesions that completely recovered at the 1-month follow-up. All patients received steroids from the day before surgery until 7 days after surgery. No wound complications were observed in any patient.

One case of fiber misplacement was probably due to technical failure. In this case, the stereotactic procedure was performed using Autoguide Medtronic and was then replaced in the same surgical session, with satisfactory outcomes using the same technique.

MRI follow-up scans were examined using the Multimodality Tumor Tracking Tool (MTTT) on the IntelliSpace Portal 9.0 to assess tumor volume changes. The images analyzed were compared to the pre-operative MRI and were obtained at 1 month in all cases and then every 3 to 5 months according to the clinical evolution and tumor type. At least three images were studied in each case.

All patients treated with LITT had a significant reduction in the volume of the tumor on the last MRI compared to pre-operative imaging. These changes were between 12 and 69%.

The overall procedure length was reduced from 9 h in the first case to 7 h in the last case. The length of stay after surgery has reduced over time; the first patient was discharged 4 days after surgery, and the last patient was discharged in the morning of the second postoperative day.

## 5. Discussion

Laser interstitial thermal therapy is a relatively new technique for treating pediatric brain tumors and is available in a limited number of centers across Europe. Data are still limited, and collecting more information is crucial for appropriate patient selection, a better understanding of its safety and risks, and integrating LITT into therapy. Pure pediatric series are scarce, contain small samples, are heterogeneous, and have short follow-up periods. Therefore, most knowledge comes from studies on adult patients and is concerned about recurrent or residual high-grade glioma (HGG) and metastatic disease (6). These studies have shown that LITT offers good results for disease control with very low risks in the long term for LGG (WHO I-II) and encouraging results for HGG (WHO III-IV) with extended survival in recurrent or residual tumors in patients who are refractory to other treatments and not suitable for reoperation (7, 8). Moreover, most treated and described pediatric cases are associated with epilepsy (86%), with oncologic cases accounting for only 16% (9). Therefore, it is inappropriate to directly transfer these results to pediatric neurooncological patients.

Another limitation in children is that depending on their age, the thickness of their skull could prevent the use of stereotactic techniques. However, this issue is seldom discussed in the pediatric medical literature dedicated to LITT even though it is a crucial aspect of the procedure. In the largest pediatric series available on LITT for pediatric brain tumors, which included 17 different North American centers, three different systems were used consistently such as the frame-based targeting system (50%), frameless system (25,6%), and robotic system (24.4%), but the reasons for selecting different systems were not explored. In our study, we used three different techniques on six patients. In the first case, the Vertek®Medtronic System was used. In case number six, the skull had an estimated thickness of 4 mm, increasing the risk of pins. In this case, we chose Navigus Medtronic®. This system is reliable for both biopsy and LITT (4, 5). We experienced a technical issue with the malposition of the laser fiber. The Autoguide Medtronic® system was used. The fiber was repositioned using the same system in the same operating session, and the procedure was completed with good outcomes (Figure 4). Malposition is already described in adults and children, mainly depending on a technical failure and should be considered a possible complication of hemorrhage, ineffective ablation, and reoperation (6, 8–12). In our series, we observed no mortality or long-term morbidity. Ahluwalia et al. (13) stated that “no unanticipated adverse events occurred” in their adult series. The complications reported have primarily been bleeding, transient or permanent deficits related to proximity to eloquent areas, and edema, with no significant differences in adults and pediatric series (3, 6–10, 13–17). We experienced two transient power worsening in two cases of thalamic tumors, with complete recovery at 1 month. We did not experience any wound problems, and these have rarely been reported, having been described in only three cases in adults in the literature (12, 18). Mortality has been reported in both adult and pediatric series. Pehlivan et al. reported one case out of 17 (14), and Arocho-Quinones reported two deaths in 86 patients (6). All three cases were related to bleeding. It is noteworthy that in a review of 303 pediatric cases, mixed for epilepsy and oncology, published before the Arocho-Quinones series (6), mortality was *nihil* (9). Death due to perilesional edema has been described in adults (12). Edema is considered the most common cause of symptoms and headache, and particular care should be taken when dealing with lesions larger than 5 cm in diameter, near functional areas, and/or in relatively closed compartments (e.g., posterior fossa), and/or if more than one catheter is used (7, 8, 12). In these cases, some authors suggest administering steroids even during preparation for surgery (19).

**Figure 8:**
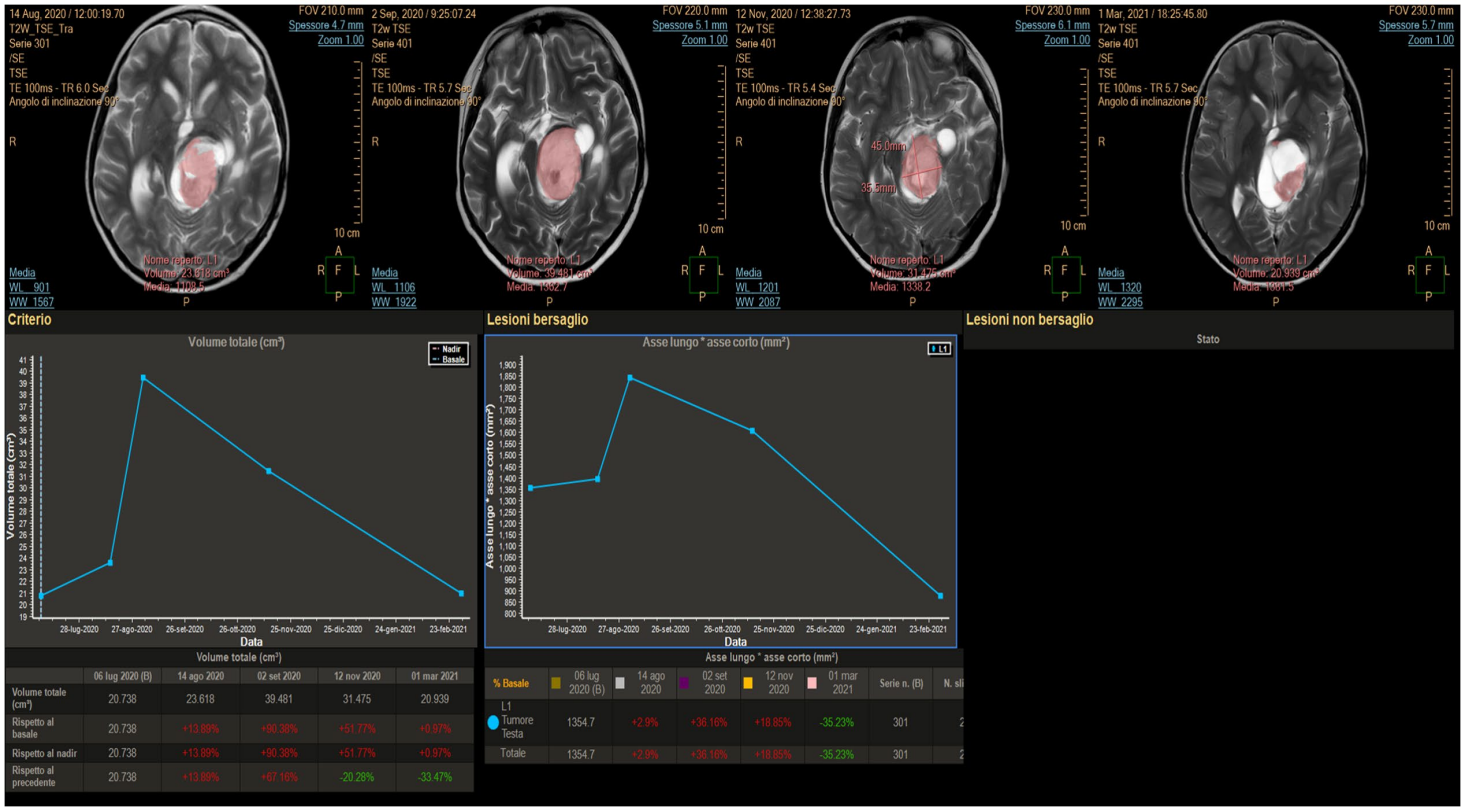
MITT Intellispace Portal imaging examination focused on the solid component of the tumor (case 2). Initially, after laser interstitial thermal therapy, the tumor appeared to increase in size due to edema, and the lesion looked different. However, a progressive reduction of the solid component by 33.5% occurred, with the ratio between the two main axes reducing by 35.2%.

To date, the most comprehensive study on LITT in pediatric brain tumors is the multi-institutional study by Arroho-Quinones that described 86 pediatric brain tumors, with ten of them being classified as high-grade, having a mean follow-up of 24 months (6). Similar to adults, their results offer a better outcome in terms of volume reduction for low-grade tumors and a higher risk for further surgery on high-grade tumors even if the data are not statistically significant. It should be noted that available data on adults and children still are not able to clarify the effect of LITT on the biology of tumors and the surrounding brain and that clinical outcomes are not completely understood (20). An interesting question regarding the interaction between LITT and chemotherapy arises. The idea of prolonged damage to the blood–brain barrier (BBB) after LITT was already evident in early reports that described ring post-contrast enhancement 6 months post-treatment (21). Further studies on mice, later confirmed in patients, demonstrated an increase in BBB permeability in molecules as large as IgG, which peaked at the third week after treatment and then gradually reduced, suggesting a possible explanation for the observed stronger favorable results after chemotherapy in patients treated with LITT (22, 23). It appears that LITT causes a disruption of tight junctions in the BBB, allowing increased access to large molecules such as doxorubicin, and a phase II study showed increased overall survival in patients treated with LITT + chemotherapy for HGG compared to the control group, with even better results in the group that started therapy later (23, 24). Hyperthermia changes cytokine and chemokine production, enhancing antigen presentation as well as cytotoxic activity of T-cells, natural killer cells, and phagocytosis (25), which constitute the immune response of the human body against the tumor. If chemotherapy is offered too early, it will probably reduce the immune response of the host against the tumor, which would explain why the results of the late-arm chemotherapy appear more relevant (24). Consistent with these preliminary and encouraging results, we observed a strong response to chemotherapy in case 6, an HGG treated with LITT. In this case, chemotherapy was commenced late unintentionally; however, the favorable results indicate that starting chemotherapy after LITT could be favorable (Figures 7C,D). The theory of immune stimulation of the host against tumors after LITT is particularly interesting. This theory is not completely new, and it was already advocated in the past to explain the reduction or even complete disappearance of tumors, especially diencephalic LGG, after partial resection or biopsies (26). We observed a significant and progressive reduction in the solid part of the tumor in case 2, an LGG that was first treated at a different institution with partial resection. After the first surgery, the tumor continued to grow. At first, after LITT, there was a significant increase in size related to edema; subsequently, the solid component continued to shrink consistently over time. As is commonly observed in pilocytic astrocytomas in similar locations, the cystic components of the tumors increased in size (27, 28), but the patient did not develop any associated signs/symptoms (Figure 8).

Radiologically, Pehlivan (14) and Arrocho-Quinones (6) described a reduction in tumor volume in approximately 80% of children treated with LITT, with complete resolution in 36% of the cases (14). Comparing the radiological results of our limited series to the literature, we can state that all six tumors treated with LITT reduced in volume over time. At follow-up, all tumors showed similar behavior. At the monthly follow-up, diffusion-weighted MRI showed a region of reduced diffusion and apparent tissue colliquation at the site where the tip of the LDF was at the time of treatment in all patients. LITT induced BBB damage in all patients because of the change in the enhancement distribution compared to the pre-operative imaging, with a typical round-shaped distribution that was still evident 1 year after the procedure in five patients. In all patients, MRI obtained at the end of the procedures indicated that the tumor had a swollen appearance related to increased edema, as documented by sequences with a long relaxation time and on ADC maps. Associated extra-lesional edema was evident in only two cases at that time (cases two and six). In all cases, edema was not evident at the 1-month follow-up, when the decrease in the overall volume started to become evident (29).

In our experience, combining biopsy with LITT in the same session is feasible and does not interfere with the results in terms of laser effectiveness. When the laser was switched on, we first used low power to understand how the heat spreads across the lesion. This was done because an apparently homogeneous lesion on MRI does not always spread the thermal wave congruously. This is probably because there is inhomogeneity in the absorption coefficient of energy in the tumoral tissue, which is supposed to be higher in abnormal tissues (30). Subsequently, the laser power is increased. The procedure is still time-consuming even if the time was reduced from 9 to 7 h between the first and last procedure. Moreover, the movement of patients between two different floors of the OR and MR suites also adds to the time taken. Patients are discharged on day two after surgery and usually do not require painkillers. Families reported that they felt the benefits of LITT were the shorter hospital stay, the lower level of permanent risk, and the possibility of proceeding with further surgery if LITT was not enough. According to the literature, Zeller reported that 77.3% of the overall described complications after LITT were transient (9). The patient selection remains unclear. We offer LITT for deep-seated tumors as we believe these have a lower surgical risk. Tumor selection could probably be extended in future to superficial lesions as the results appear promising (31). Eventually, in very young children and children with complex lesions (e.g., diencephalic tumors), LITT can be included as part of a multistep strategy to delay radiotherapy. The increased effect of chemotherapy after LITT is very promising; further studies are needed to confirm this finding.

Our experience, even with limited time and number of patients, focuses mainly on deep-seated lesions, in the thalamus and periventricular and peduncular locations. We choose to offer LITT to patients where surgical risks may be significant, while we continue to offer traditional open surgery to more superficial tumors (e.g., lobar tumor). The biggest available series on LITT in pediatric brain tumors were reported by Arocho-Quinones et al. (6) describing 86 cases from 17 North American centers, with a relatively short mean follow-up of 24 months, divided the tumors according to the locations in lobar, deep-seated and cerebellar. Even if the group of the deep-seated tumors accounted for 39 patients, harboring lesions located in the thalamus, hypothalamus, basal ganglia, and periventricular region, there is no specific description of results, indication, and complications for this group. According to that study, tumor location does not significantly change the risk of complications associated with LITT. They focus results mainly on volume changes and offer data on 72 patients out of 86 with a better outcome of reduction or stability of the tumor in 83.1% of LGG and 57.1% of HGG.

In our experience, LITT offered some relevant advantages over open surgery. It was an overall safe procedure and minimally invasive. Hospitalization length was shorter, harboring a positive effect on the psychological impact on the patients and the caregivers, and the risk of infection and wound complications were *nihil* in our series and is found very rarely in the literature as well. We observed a reduction of the volume of the tumor in all the patients with the exception of one LGG that experienced regrowth after 18 months from the treatment. The overall length of the procedure and its overall cost were probably the strongest disadvantages to LITT. Similar observations are reported in the systematic review on pediatric LITT from Zeller et al. (9). Large prospective studies with long follow-up and comparative studies confronting open surgery to LITT would significantly help to clarify indications, risks, and results of LITT in pediatric brain tumor.

## 6. Conclusion

Our data and the existing literature suggest that LITT can be considered an effective tool in the surgical treatment of pediatric brain tumors. The use of the stereotactic technique can be challenging in children compared to adults. The safety profile is acceptable, especially if the overall treated volume is limited, invasiveness is strongly reduced compared with standard surgery, and deep lesions located in functional areas can be reached. LITT can be part of a multistep treatment protocol and can be repeated. It is effective in different histologies with probably better outcomes in low-grade tumors. Preliminary studies have suggested that chemotherapy is more efficacious if administered after LITT. Further studies are necessary to enhance our understanding of the performance of LITT in children.

## Data availability statement

The raw data supporting the conclusions of this article will be made available by the authors, without undue reservation.

## Ethics statement

Written informed consent was obtained from the minor(s)’ legal guardian/next of kin for the publication of any potentially identifiable images or data included in this article.

## Author contributions

BS: draft and writing. MM: writing, imaging selection, and elaboration. MG: data collection. ST: imaging collection and imaging elaboration. LG: draft and revision. All authors contributed to the article and approved the submitted version.

## Funding

The authors acknowledge that funding for open access was provided from Fondazione Meyer and La forza di Giò-ODV.

## Conflict of interest

The authors declare that the research was conducted in the absence of any commercial or financial relationships that could be construed as a potential conflict of interest.

## Publisher’s note

All claims expressed in this article are solely those of the authors and do not necessarily represent those of their affiliated organizations, or those of the publisher, the editors and the reviewers. Any product that may be evaluated in this article, or claim that may be made by its manufacturer, is not guaranteed or endorsed by the publisher.
